# Dietary Gluten-Free Regimen Does Not Affect the Suppression of the Inflammatory Response in the Arachidonic Acid Cascade in Hashimoto’s Disease

**DOI:** 10.3390/ijms26136507

**Published:** 2025-07-06

**Authors:** Małgorzata Szczuko, Lidia Kwiatkowska, Urszula Szczuko, Leon Rudak, Karina Ryterska, Anhelli Syrenicz, Jakub Pobłocki, Arleta Drozd

**Affiliations:** 1 Department of Human Nutrition and Metabolomics, Pomeranian Medical University in Szczecin, 71-460 Szczecin, Polandarleta.drozd@pum.edu.pl (A.D.); 2 Department of Bromatology and Nutritional Diagnostics, Pomeranian Medical University in Szczecin, 71-460 Szczecin, Poland; 3 Department of Endocrinology, Metabolic Diseases and Internal Diseases, Pomeranian Medical University, 70-252 Szczecin, Poland; anhelli.syrenicz@pum.edu.pl (A.S.); jakub.poblocki@pum.edu.pl (J.P.)

**Keywords:** Hashimoto’s disease, GFD, arachidonic acid, thromboxane B2, prostaglandin E2, leukotriene B4, 16-RS HETE

## Abstract

The incidence of Hashimoto’s disease (HD) increases with age and in people who have other autoimmune diseases. It is characterized by lymphocytic infiltration, fibrosis, and atrophy of the thyroid parenchyma with the simultaneous presence of thyroid peroxidase antibodies (ATPO) and/or thyroglobulin antibodies (ATG). Eicosanoids are formed via the cyclooxygenase (COX), lipoxygenase (LOX), and monooxygenase (CYP450) pathways with arachidonic acid (ARA), resulting in the production of epoxyeicosatrienoic acids (EETs) or hydroxyeicosatetraenoic acids (HETEs). These eicosanoids can act in an autocrine or paracrine manner on target cells. This study aimed to examine whether a gluten-free diet (GFD) can modulate the enzymatic pathways of the pro-inflammatory ARA cascade. The study material consisted of serum samples from Caucasian female patients with HD aged 18–55 years. Participants were enrolled in the study based on the presence of an ultrasound characteristic of HD, and elevated serum levels of anti-thyroid peroxidase antibodies and anti-thyroglobulin antibodies. Patients with confirmed celiac disease did not participate in the study. A total of 78 samples were analyzed, with 39 collected after 3 months of following a GFD. Eicosanoids (thromboxane B2, prostaglandin E2, leukotriene B4, and 16R-hydroxy-5Z,8Z,11Z,14Z-eicosatetraenoic acid (16-RS HETE)) were extracted using high-performance liquid chromatography. The contribution of leukotriene (LTB) was analyzed in the LOX pathway, prostaglandins (PGE2) and thromboxane (TXB2) were selected for the involvement of the COX pathway, and 16RS HETE was used for the CYP450 pathway. All parameters were analyzed before and after a 3-month dietary intervention that included a gluten-free diet. In the obtained results, only one mediator, leukotriene B4, was significant (*p* < 0.05). The mean level on the initial visit was 0.202 ± 0.11 (SD), while it was 0.421 ± 0.27 (SD) on the subsequent visit, indicating a significant increase in its level after implementing a GFD. Although there was a trend in the CYP 450 pathway of decreased 16-RS HETE, the presented correlations show that thromboxane B4 and 16RS-HETE were positively correlated with the body mass and body fat mass of the examined patients. There was a trend in the CYP 450 pathway of decreased 16-RS HETE after GFD. Thromboxane B4 and 16RS-HETE levels before GFD were positively correlated with the body mass and body fat mass of the examined patients. A gluten-free diet in HD does not suppress the synthetic pathways of LOX, COX, or cytochrome P450 (CYP450). The level of adipose tissue has a greater impact on the inflammatory processes in HD than a gluten-free diet. This study does not confirm the suppressive effect of a gluten-free diet on the pro-inflammatory arachidonic acid cascade in any of the three analyzed mediator synthesis LOX, COX, CYP450 pathways.

## 1. Introduction

Hashimoto’s disease (HD), also known as Hashimoto’s thyroiditis, is an autoimmune thyroid disorder [1]. The disease is detected ten times less frequently in men than in women [2]. The White ethnic group is characterized by a higher prevalence of the disease compared with Black or Asian ethnic groups. Common coexisting conditions include other autoimmune diseases, such as celiac disease, myasthenia gravis, Sjögren’s syndrome, autoimmune liver disease, pernicious anemia, and systemic sclerosis. Less commonly, other endocrinopathies are associated with the disease, referred to as APSs (autoimmune polyendocrine syndromes). Advanced Hashimoto’s disease is associated with overt hypothyroidism, manifested by elevated TSH levels, decreased free thyroxine (fT4) levels, and sometimes decreased free triiodothyronine (fT3) levels. However, most patients have a subclinical type of disease [3].

**Figure 1:**
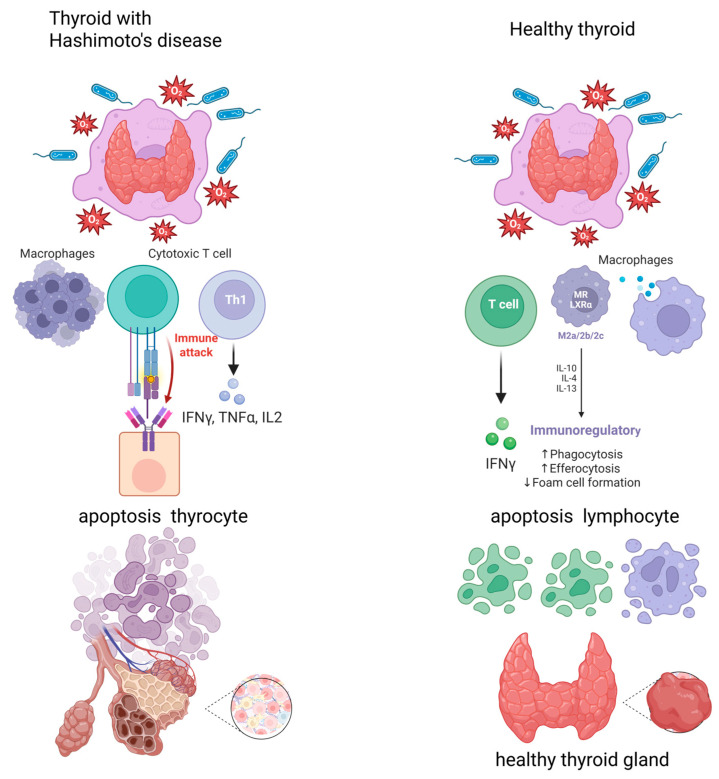
Apoptosis with the participation of T cells in Hashimoto’s thyroiditis and in a healthy thyroid gland. Created with BioRender.com/app.biorender.com, accessed on 4 July 2025.

The etiology of HD has not been fully explained, but it involves genetic, epigenetic, and environmental factors that interact. Several responsible polymorphisms concerning genetic factors have been identified, including those in the HLA tissue compatibility system, immune-regulating genes such as *CTLA-4*, *CD40*, *FOXP3*, *CD25*, and *PTPN22*, and thyroid-specific genes [1,4]. Environmental factors include stress, pregnancy, cytokine therapies, certain drugs such as amiodarone or lithium salts, and a diet excessively rich in iodine [2,3]. Autoimmune reactions can also be exacerbated by selenium deficiency, smoking, or infection with the hepatitis C virus [2,5]. HD involves the gradual atrophy of the thyroid gland, which is a consequence of the infiltration of lymphocytic cells, follicular atrophy, and congestion with the metaplasia of follicular cells [6]. In the course of HD, autoreactive T lymphocytes are generated either through suppression by regulatory CD25+CD4+ and CD8+CD122+ T lymphocytes or due to abnormal MHC class II complex expression on thyroid cell surfaces. Stimulated CD4+ T lymphocytes activate cytotoxic CD8+ lymphocytes and B lymphocytes, which are subsequently transformed into plasma cells that produce antibodies. These antibodies destroy the thyroid gland, leading to the gradual development of hypothyroidism, although it has a subclinical form in most patients. After binding to follicular cells, lymphocytes release their highly lytic content, leading to the apoptosis of thyroid cells [7]. In autoimmune thyroiditis, activated macrophages and Th1-dominant T lymphocytes are present. They produce IL-2, IFN-γ, and TNF-α, with a relative absence of IL-4 or -10, suggesting that these cells mainly support the induction of apoptosis by T cells [8]. A schematic representation of the described process is shown in Figure 1.

Arachidonic acid (AA), also known as eicosatetraenoic acid, is a polyunsaturated fatty acid (PUFA) ω-6. It is primarily found in the form of phospholipids in cell membranes [9,10]. It is one of the most abundant polyunsaturated fatty acids in the human body and serves as a substrate for numerous enzymatic processes that produce bioactive lipid mediators such as leukotrienes, prostaglandins, and epoxyeicosatetraenoic acids. When cells are subjected to stress, these acids are released from phospholipids by phospholipase A2 and phospholipase C into free arachidonic acids, which serve as precursors for pro-inflammatory mediators produced via the three synthesis pathways described below [11,12].

The cyclooxygenase pathway catalyzes the formation of prostaglandins (PGs) and thromboxane (TX) [13]. Leukotrienes and lipoxins are produced via the action of lipoxygenase enzymes [9,10]. Lipoxygenase enzymes (5-LOX, 8-LOX, 12-LOX, and 15-LOX) convert AA into hydroxyperoxyeicosatetraenoic acids (HPETE), which are subsequently reduced to monohydroxyeicosatetraenoic acids (HETE). These compounds further transform into biologically active compounds such as leukotriene A4 (LTA4), LTC4, LTD4, LTE4, and LTB4, as well as lipoxins LXA4 and LXB4 [13,14]. In addition to cyclooxygenase or lipoxygenase activity, cytochrome P450 oxidases are involved in the third process of oxidizing specific PUFAs. These enzymes produce epoxyeicosatrienoic acids (EET) or hydroxyeicosatetraenoic acids (HETE) [9,10].

Gluten is defined as a protein fraction derived from wheat, rye, barley, oats, or their crossbred varieties and their derivatives. From a chemical standpoint, gluten is a protein composed of glutelin and prolamin fractions [15,16,17]. Gliadin and glutenin are the prolamin and glutelin fractions of wheat, while secalin, hordein, and avenin comprise the respective prolamin fractions of rye, barley, and oats. Sections of gluten proteins are not fully degraded by human digestive enzymes, leading them to pass into the small intestine. According to European regulations, the total gluten content should be less than 20 ppm per day [17]. It is worth noting that increased gluten consumption raises the risk of non-celiac gluten sensitivity and celiac disease in the population [18].

This study aimed to present the results of research on the impact of a three month-gluten-free diet used by 39 Caucasian female patients aged 18–55 years with HD on the activity of three pro-inflammatory mediator synthetic pathways.

## 2. Results

### 2.1. Characteristics of Study Group

The average age of the patients was 37.91 ± 8.97, BMI was 25.47 ± 4.29, and the content of adipose tissue was 35.68 ± 7.4%. Excess adipose tissue was found in all patients participating in the study (>25%). The socio-demographic, anthropometric, and biochemical data of the study group are presented in Table 1. Patient parameters were compared before and after implementing a gluten-free diet (GFD). The data indicate no significant differences, except for the TSH levels before and after the diet and concomitant treatment of thyroid dysfunction.

BMI (>25) and especially body fat content (<30%) were increased (Table 1). The levels of both tested antibodies, ATPO and ATG, were elevated. Before the GFD, the mean ATPO and ATG levels were 187.879 ± 137.993 IU/mL and 318.946 ± 562.052 IU/mL, respectively, while the medians were 138.05 and 181.02 IU/mL. After the GFD, the mean ATPO and ATG levels were 197.51 ± 233.735 IU/mL and 283.35 ± 478.578 IU/mL, respectively, while the medians were 165.7 and 211.08 IU/mL. The remaining parameters shown in Table 1 had a normal distribution and were within the reference range.

### 2.2. Pro-Inflammatory Derivatives of Arachidonic Acid

By analyzing the measurements of selected pro-inflammatory mediators of arachidonic acid, it was found that the mean levels of TXB2 in the group before and after the diet were 1.42 ± 2.19 µg/mL and 1.55 ± 1.77 µg/mL, respectively, and the difference was not statistically significant (*p* = 0.763). Furthermore, the respective levels of PGE2 were 8.4 ± 9.9 µg/mL and 8.66 ± 9.21 µg/mL; those of LTB4 were 0.2 ± 0.11 µg/mL and 0.42 ± 0.27 µg/mL; and those of 16RS-HETE were 5.36 ± 3.34 µg/mL and 4.01 ± 2.65 µg/mL. Significant differences were observed between the groups for LTB4 and 16RS-HETE before and after the diet, whereas the results for TXB2 and PGE2 did not differ significantly, as presented in Table 2.

The next stage of the planned research was to check the relationships between metabolites and anthropometric and biochemical parameters before and after using a gluten-free diet, the results of which are presented in detail in Table 3. Positive correlations between TXB2 and body weight and fat tissue content before the diet were found. A positive correlation between leukotriene and CRP was also observed before and after the diet. Positive correlations between 16RS-HETE and body weight and fat tissue mass before the introduction of a gluten-free diet were also observed. No other significant correlations with other parameters were found. The results are presented in Table 3.

### 2.3. Summary of Results

- Using a gluten-free diet for three months did not lead to a statistically significant change in the anthropometric and biochemical parameters, except for TSH (which resulted from concomitant levothyroxine treatment in the patients).
- The levels of mediators from the lipoxygenase pathway (LTB4) increased statistically significantly after gluten elimination, and the monooxygenase pathway (16RS-HETE) decreased after gluten elimination from the diet (trend).
- There was a positive correlation among TXB2, LTB4, and body weight before and after three months of following a gluten-free diet.
- There was a positive correlation among 16RS-HETE, body weight, and body fat mass before implementing a gluten-free diet.
- LTB4 was significantly correlated with CRP before and after following a gluten-free diet.

## 3. Discussion

There is conflicting evidence regarding whether early diagnosis and adherence to a GFD can slow the progression of autoimmune diseases. According to the current literature, HD and celiac disease sometimes coexist (5%) more often than in other patient groups [19,20]. They share a similar genetic background [21]. Antibodies against tissue transglutaminase, whose levels are elevated in celiac disease, can react with transglutaminase present in the thyroid gland and participate in the pathogenesis of HD. However, this mechanism is impossible when HT does not coexist with celiac disease. Therefore, it can be concluded that using a gluten-free diet in primary HT prevention is unwarranted [22]. The rationale for using this diet is only in cases where HT and celiac disease or other forms of gluten intolerance coexist [23]. For the above reasons, it is advisable to screen all individuals with HT for celiac disease to rule it out. Serological tests could be conducted tik determine the presence of IgA and IgG antibodies against gliadin, anti-transglutaminase IgA antibodies (TGAs), and anti-endomysium IgA antibodies in serum (EMAs). In the case of a positive result for any of these, patients are referred for gastroduodenoscopy and duodenal biopsy. It should be noted that the presence of antibodies in general does not necessarily reflect a clinical autoimmune disease [23].

Sategna-Guidetti’s studies showed that 12.9% of patients achieved the normalization of subclinical hypothyroidism by adopting a gluten-free diet in celiac patients [24]. It was found that impaired levothyroxine absorption during hypothyroidism treatment should lead doctors to suspect celiac disease [24]. In Ventura’s study, a beneficial effect of gluten elimination from the diet in the course of HT was presented. It was suggested that eliminating gluten from the diet in patients with celiac disease coexisting with HT may reduce the number of ATG and ATPO antibodies [25]. However, we did not observe such significance in our own research. Meanwhile, Malandrini pointed out the lack of a connection between a gluten-free diet and HD, as they did not confirm a relationship between the titers of thyroid antibodies and the use of a gluten-free diet [26]. This study corresponds with our own results. A study by Carrocio et al., which examined individuals suffering from non-celiac gluten sensitivity (NCGS), showed that the frequency of autoimmune diseases in these patients was significantly higher than in the control group (*p* < 0.001) [27]. HD was the most frequently reported autoimmune disease associated with the presence of IgG antibodies against gliadin. The study also found the presence of antinuclear antibodies (ANAs, markers of autoimmune diseases) in 46% of NCGS patients compared with 2% of patients without NCGS [26]. Therefore, it can be concluded that patients with forms of gluten intolerance are significantly more likely to develop autoimmune diseases, especially HT, than individuals without gluten tolerance issues. Moreover patients with immunological reactivity to food proteins of the IgG1–3 class were more likely to have tissue antibodies present than patients in whom food antibodies were not identified. In individuals with positive serum antibodies against gliadin, as many as 64% developed antibodies against their tissue antigens compared with 35% of patients without antibodies against gliadin [28]. The studies carried out by Riseh et al. confirmed the increased risk of celiac disease in patients with HD and the frequent occurrence of its asymptomatic form in this group of patients [29]. It was also found that gluten consumption can impact the composition of the gut microbiota, which may lead to dysbiosis. Dysbiosis can strengthen the vicious cycle of intestinal epithelial damage, chronic inflammation and, in individuals with a genetic predisposition, autoimmunization [21].

Celiac disease can reduce iodine absorption, which can complicate the treatment of HD. Reducing the levels of anti-thyroid antibodies may limit autoimmune processes and have a beneficial effect on the course of HD in individuals with concurrent celiac disease and NCGS.

As the participation of some eicosanoids in autoimmune diseases (autoimmune diabetes, rheumatoid arthritis) has already been indicated in a few studies in this area, we decided to examine their participation in HD [30,31]. No studies of the LOX, COX, or CYP450 pathways in HD were found, and very few on the effect of the GFD diet on these pathways. Therefore, it is not possible to compare our own results with the literature.

The analysis of our results showed that the leukotriene B4 level increased after implementing a gluten-free diet in patients with HD. This may indicate a potentially ongoing inflammatory process, and the gluten-free diet did not have the expected impact upon it. Although our study was conducted on a relatively small patient cohort, which constitutes a limitation, it represents a pioneering effort in this area. Based on the results, we hypothesize that the influence of a gluten-free diet in HD is minimal, and any observed beneficial effects may be attributed to accompanying dietary modifications—such as increased vegetable intake and supplementation with omega-3 and omega-6 fatty acids—rather than gluten elimination itself. These findings are consistent with our prior long-term observations (12 months). In the present study, the intervention period was reduced to 3 months to ensure stricter adherence, yet no measurable effect was observed. Based on our research, it appears that the level of mediators such as LTB4 significantly increased after following the GFD, indicating that the diet did not have a calming effect on inflammatory processes. However, the level of 16RS-HETE in the CYP 450 pathway decreased slightly, therefore further research in this direction is justified. Positive correlations were also observed between these mediators and body mass and fat tissue content, demonstrating the active involvement of these anthropometric factors in inflammatory processes in the body. No other studies on the levels of mediators in the cyclooxygenase and lipoxygenase pathways in individuals with a higher body mass and fat tissue content have been found. A positive relationship was found between body mass and TXB2 levels before and after the gluten-free diet, suggesting the diet did not influence the TXB2 levels, and the mediator was independently associated.

Thyroxine (levothyroxine, LT4) remains the standard therapy for hypothyroidism resulting from HD, effectively restoring euthyroid status in the majority of patients. However, its influence on underlying autoimmune and inflammatory activity remains a subject of ongoing investigation [32]. Several studies suggest that LT4 treatment may exert modest anti-inflammatory effects, potentially through negative feedback on TSH levels, which, in turn, can modulate immune cell activity within the thyroid gland. The suppression of TSH has been associated with reduced lymphocytic infiltration and the decreased production of pro-inflammatory cytokines such as interleukin-6 (IL-6) and tumor necrosis factor-alpha (TNF-α) [33]. Nonetheless, levothyroxine is not considered an immunomodulatory treatment per se, and its capacity to reverse chronic inflammation or halt autoimmune progression is limited. In our study, participants were euthyroid at baseline due to ongoing LT4 therapy, which may have attenuated detectable inflammatory responses, thus influencing the interpretation of immune and metabolic markers, including those associated with the LOX, COX, and CYP450 pathways. This may be a limitation of the study; therefore, in future studies, patient selection may be required.

Currently, there is no evidence to explain the mechanism responsible for the positive impact of a GFD on thyroid autoimmunity. However, a gluten-free diet can have an anti-inflammatory effect independently, especially if gluten-containing grain products are replaced with fresh vegetables and berries. Unlike a conventional diet, which modifies cytokines toward an inflammatory profile, proper adherence to a GFD rich in antioxidants can reduce inflammation. Instead of a conventional diet rich in fats and processed foods, introducing guidelines for the consumption of anti-inflammatory components such as omega-3 fatty acids—including EPA and DHA—as well as antioxidant polyphenols and vitamins may be helpful in the therapy of Hashimoto’s disease.

## 4. Materials and Methods

### 4.1. Study Group

This study involved 39 Caucasian female patients with HD from whom serum samples were collected before and after 3 months of following a gluten-free diet (FDA). Patients with confirmed celiac disease did not participate in the study. Height and body weight were recorded with an accuracy of 0.5 cm and 0.1 kg, respectively. Body tissue composition was assessed using Dual Energy X-ray Absorptiometry (DXA) on a GE Lunar Prodigy Advance (Madison, WI, USA). Participants were enrolled in the study based on the presence of an ultrasound pattern characteristic of HD, assessed using the ALOKA Prosound Alpha-7 system with a linear UST-5411 probe of 4.4–13.3 MHz (Tokyo, Japan), along with elevated serum levels of anti-thyroid peroxidase antibodies (ATPO > 34 IU/mL) and/or anti-thyroglobulin antibodies (ATG > 115 IU/mL). Thyroid function status was evaluated by measuring serum concentrations of thyroid-stimulating hormone (TSH; reference range 0.270–4.200 µIU/mL), free thyroxine (fT4; 0.93–1.70 ng/dL), and free triiodothyronine (fT3; 2.00–4.40 pg/mL). All study participants were euthyroid at the time of enrollment (the range of thyroxine dose used was 25–150 µg/day). The group consisted of newly diagnosed (*n* = 19) patients and those on chronic treatment (*n* = 20). Fasting blood samples were collected in polypropylene tubes containing EDTA, then centrifuged at 3000 rpm for 10 min in a refrigerated centrifuge. The separated plasma was transferred into Eppendorf tubes and stored at −80 °C until analysis. The extended thyroid profile in serum levels (TSH, fT3, fT4, ATPO, and ATG antibodies) were measured in all subjects using the electrochemiluminescence immunoassay (ECLIA) method on a Roche Cobas 6000 analyzer, module 601 (Indianapolis, IN, USA).

### 4.2. Dietary Intervention

Previous use of a gluten-free diet, malabsorption syndromes, intestinal or gastric resection, thyroidectomy, Graves–Basedow’s disease, as well as coronary artery disease, diabetes, hypertension and use of glucocorticosteroids, nonsteroidal anti-inflammatory drugs, immunosuppressive drugs, or drugs affecting the thyroid axis other than levothyroxine were the exclusion criteria. All participants were provided with nutritional guidance along with a sample gluten-free diet (GFD) plan totaling 1800–2000 kcal. The dietary intervention was maintained for a minimum duration of six months. Inclusion in the GFD study was limited to individuals who demonstrated excellent (*n* = 18), good (*n* = 13), or satisfactory (*n* = 8) compliance with dietary recommendations, as assessed using a dedicated adherence questionnaire [34].

### 4.3. Eicosanoid Extraction

Substances including 16RS-HETE, LTB4, PGE2, and TXB2 were extracted from serum using RP-18 SPE columns (Agilent Technologies, Cheadle, UK). For the eicosanoid extraction, 0.5 mL of serum was added to 1 mL of acetonitrile to precipitate proteins along with 50 μL (1 μg/mL) of an internal standard. After 15 min of incubation at −20 °C, the samples were centrifuged at 10,000 rpm for 10 min using a cooled centrifuge (Eppendorf, Centrifuge 5804R, Hamburg, Germany). The supernatants were transferred to new collection tubes, and 4.5 mL of 1 mM HCl was added. The pH of each sample was adjusted to 3 by adding 30–50 μL of 1 M HCl. The columns were activated through successive washes with 3 mL of 100% acetonitrile and 3 mL of 20% acetonitrile in water. The samples were loaded and washed twice with 3 mL of 20% acetonitrile in water. Eicosanoids were further eluted using a 1.5 mL mixture of methanol and ethyl acetate (1/1 *v*/*v*), dried under vacuum, and reconstituted in 100 μL of 60% methanol in water with the addition of 0.1% acetic acid. The samples were immediately analyzed using HPLC [19]. Leukotriene B4 (LTB4) was assessed as a marker of the lipoxygenase (LOX) pathway, while prostaglandin E2 (PGE2) and thromboxane B2 (TXB2) were analyzed to evaluate cyclooxygenase (COX) pathway activity. For the cytochrome P450 (CYP450) pathway, 16RS-HETE was used as the representative metabolite.

### 4.4. High-Performance Liquid Chromatography (HPLC)

The HPLC operating parameters were as follows: the HPLC separations were conducted using an Agilent Technologies 1.260 liquid chromatograph consisting of a degasser (model G1379B), binary pump (model G1312B), column oven (model G1316A), and diode array detector (model G1315CDAD VL+). Sample injections were performed using a G1329B. Agilent ChemStation software version 2.8. (Agilent Technologies, Cheadle, UK) was utilized for instrument control, data acquisition, and analysis. Separation was achieved using a Thermo Scientific Hypersil BDS C18 column (100 × 4.6 Mm, 2.4 μm; catalog number 28102-154630) with the column oven temperature set at 20 °C. Free fatty acid (FFA) levels, expressed in µg/mL, were converted to the FFA percentage in the serum. A gradient method was employed, with the mobile phase consisting of a mixture of solvent A (methanol/water/acetic acid, 50/50/0.1, *v*/*v*/*v*) and solvent B (methanol/water/acetic acid, 100/0/0.1, *v*/*v*/*v*). The buffer B content in the mobile phase was 30% from 0 to 2 min of separation, linearly increased to 80% over 33 min, held at 98% from 33.1 to 37.5 min, and returned to 30% from 40.3 to 45 min. The flow rate was set at 1.0 mL/min, and the sample injection volume was 60 µL. The diode array detector (DAD) monitored absorption peaks at 280 nm for LTB4 and 210 nm for PGE2, 16RS-HETE, and TXB2 [35]. The absorbance spectra of the peaks were analyzed to confirm the analyte identification. Quantification was based on peak areas with internal standard calibration. Quantitative analysis was performed using ChemStation software (Agilent Technologies, Cheadle, UK) [35,36,37].

### 4.5. Statistical Analysis

Statistical analyses were conducted using Statistica 13.3 software (Statsoft, Krakow, Poland). The data distribution was assessed using the Shapiro–Wilk test, and it was found that the results had a distribution other than normal. Then, the data were analyzed using Spearman’s rank correlation. Results were considered statistically significant at *p* < 0.05.

## 5. Conclusions

The results of this research indicate that a gluten-free diet used in Hashimoto’s thyroiditis does not suppress the lipoxygenase (LTB4), cyclooxygenase (TXB2), or cytochrome P450 monooxygenase (16RS-HETE) synthetic pathways.

The relationship between LTB4 and TXB2 and body mass before and after the intervention indicates that excess body fat has a greater influence on the inflammatory response in HD than the GFD. Therefore, maintaining a healthy body weight and fat tissue level is a priority. A similar effect was observed in the case of the cytochrome P450 monooxygenase pathway (16RS-HETE).

The correlation between LTB4 and CRP before and after the gluten-free diet intervention confirms the lack of the expected effect of the diet in terms of dampening the inflammatory response involving acute-phase proteins.

Due to its restrictive nature, a gluten-free diet for HD patients may pose more risks than benefits, as it can lead to nutritional deficiencies and excess fat in the diet. This research did not confirm the calming effect of the gluten-free diet on the arachidonic acid proinflammatory cascade.

## Figures and Tables

**Table 1:** Comparison of age and anthropometric and biochemical measurements before and after implementing a gluten-free diet.

Parameters	Before the Diet		After the Diet		*p*-Value
	Mean	SD	Mean	SD	
Age (years)	37.472	7.966	37.911	8.966	0.804
Height (cm)	167.085	4.749	166.842	5.989	0.814
Body weight (kg)	69.734	12.083	71.036	12.285	0.456
BMI (kg/m^2^)	25.418	3.858	25.472	4.286	0.923
Body fat mass (kg)	25.131	7.574	26.015	8.793	0.543
% body fat	34.550	5.836	35.677	7.397	0.390
ATPO (IU/mL)	187.879	137.993	197.512	233.735	0.803
ATG (IU/mL)	318.946	562.052	283.350	478.578	0.243
TSH (µIU/mL)	3.222	2.425	1.793	1.211	0.005 *
fT3 (pg/mL)	2.921	0.508	2.884	0.426	0.690
fT4 (ng/dL)	2.902	144.954	1.352	0.208	0.426
CRP (mg/L)	1.570	1.450	1.430	1.060	0.568

*—statistically significant value; ATPO—anti-thyroid peroxidase antibodies; ATG—antithyroglobulin antibodies; TSH—thyroid-stimulating hormone; fT3—free triiodothyronine; fT4—free thyroxine; CRP—C-reactive protein.

**Table 2:** Average contents of the analyzed metabolites before and after the gluten-free diet.

Mediators (µg/mL)	Before the Diet		After the Diet		*p*-Value
	Mean ± SD	Median	Mean ± SD	Median	
TXB2	1.417 ± 2.193	0.610	1.553 ± 1.773	1.223	0.764
PGE2	8.400 ± 9.901	5.612	8.661 ± 9.216	5.552	0.904
LTB4	0.202 ± 0.112	0.176	0.421 ± 0.273	0.343	0.0000 *
16RS-HETE	5.361 ± 3.349	4.245	4.019 ± 2.657	3.643	0.054 **

*—statistically significant value; **—visible relationship trend; TXB2—thromboxane; PGE2—prostaglandin; LTB4—leukotriene; 16RS-HETE—16-hydroxyeicosatetraenoic acids.

**Table 3:** Correlations between fatty acid derivatives and anthropometric and biochemical parameters before and after a gluten-free diet.

	Before the Diet			
	TXB2	PGE2	LTB4	16RS HETE
Body weight (kg)	0.338596	−0.202877	−0.011094	0.343364
BMI (kg/m^2^)	0.260730	−0.150895	0.016461	0.248190
Body fat mass (g)	0.340965	−0.224413	0.052039	0.335862
% body fat	0.249897	−0.230690	0.089829	0.281437
ATPO (IU/mL)	0.012256	0.034259	−0.136327	−0.196726
ATG (IU/mL)	0.218033	0.149694	−0.035316	0.168946
TSH (uIU/mL)	−0.201970	0.071220	−0.107853	−0.225742
fT3 (pg/mL)	−0.048557	−0.138112	−0.099590	−0.186665
fT4 (ng/dL)	0.253603	−0.059071	0.053083	0.075085
CRP (mg/L)	0.042549	0.1701	0.395769	0.111715
	**After the Diet**			
	**TXB2**	**PGE2**	**LTB4**	**16RS HETE**
Body weight (kg)	0.044887	−0.192943	−0.043332	−0.067551
BMI (kg/m^2^)	−0.060214	−0.125306	0.089704	−0.037661
Body fat mass (g)	−0.007947	−0.144111	0.032910	−0.064960
% body fat	−0.048196	−0.073610	0.104520	−0.031625
ATPO (IU/mL)	0.248300	−0.050716	−0.038358	0.021351
ATG (IU/mL)	−0.090165	−0.070293	−0.173284	−0.160617
TSH (uIU/mL)	−0.142625	−0.155101	−0.248174	−0.119689
fT3 (pg/mL)	0.124574	0.040813	0.091432	0.044906
fT4 (ng/dL)	−0.039495	−0.088208	0.011553	−0.218691
CRP (mg/L)	−0.04035	0.278355	0.641592	−0.09011

Red color—statistically significant correlations; ATPO—thyroid peroxidase antibodies; ATG—anti- thyroglobulin antibodies; TSH—thyroid-stimulating hormone; fT3—free triiodothyronine; fT4—free thyroxine; CRP—C-reactive protein.

## Data Availability

The data presented in this study are available on request from the corresponding author.
